# Zinc vs. Babbitt Metal

**Published:** 1882-09

**Authors:** 


### ZINC vs. BABBITT METAL.

Thirty years ago a partner of mine, Dr. D. H. Goodno, after experimenting with all the metals ever used for dental dies, and finding nothing satisfactory, at last thought that "Babbitt metal," then but little known, might answer the purpose. He tried it, and found, to his great relief, that it was just what was needed. We adopted it at once, and after having used it exclusively all these years, can say it is a *perfect thing* for the purpose. And why should it not be? It has all the requirements needed, viz.: *non-shrinking*, *hardness*, *toughness*, smoothness, and melting at a low temperature. Now, while *zinc* is *hard*, its shrinkage is a serious objection. *Type metal* does not shrink, but is too brittle.

But it is necessary that the Babbitt metal should be made from a correct formula. Much that is sold, while it answers the purpose for which it is generally used, viz.: bearings for machinery, is made in such a manner that it is too soft for dies. Metal that costs less than forty cents per pound will not answer, for it can't be made and sold for less.

To ensure a good article, make it youself, as follows:

Copper, 1 part;
Antimony, 2 parts;
Tin, 8 parts.

Melt in a crucible, *in the order named*, turning off as soon as the tin is dropped in, and remelt. The S. S. White Co., is now making from the above formula.

For counter die, use seven parts lead and one part tin, and don't turn too hot; coat the die with whiting.

For convenience, moisten the sand with sweet oil, as it is then always ready for use, and there will be no danger of your cast being spoiled from excess of moisture.

I seldom make the second die, even in sharp, irregular, lower cases. The plate will *always* fit the plaster model, and if that is correct, will, of course, fit the mouth. It is time that zinc was banished from dental laboratories, books of instruction and colleges. There is no more need of it than of the "fifth wheel to a coach." On the other hand, it is simply a nuisance, as any one will say after following the above directions half a dozen times. L. P. HASKELL, Chicago, Ill

To PREVENT nausea when taking impressions let the patient rinse the mouth thoroughly with camphor and water; five drops of spirits of camphor to a wine-glass of water is sufficient in most cases. Have used it so long and so successfully that we supposed everybody used it until a few days since, when in conversation with several dentists we learned that no one of the number had ever tried it. One suggested that "it perhaps gave the mucous membrane something else to think of for the time being."

WHAT I am told about mechanical and chemical action and compatibility is no object for observation for me; neither of these expressions is correct. *So long as a tooth is alive the foreign body that comes in contact with dentine will irritate.* As to chemical action, I do not know whether such a thing does exist in the relation between the dentine and the filling material. Where there is plenty of living matter, as in deciduous teeth, the irritation upon a solid filling will be more intense than upon a filling of soft, protective material. I recommend light filling material in deciduous teeth, for the reason that we have more living matter in them. HEITZMANN.

